# The association between mental health and Bulimia Nervosa among a sample of Lebanese young adults: the indirect effect of difficulties in emotion regulation

**DOI:** 10.1186/s12888-023-04847-0

**Published:** 2023-05-12

**Authors:** Reine Azzi, Serena Samaha, Diana Malaeb, Marwan Akel, Vanessa Azzi, Souheil Hallit, Sahar Obeid

**Affiliations:** 1 School of Medicine and Medical Sciences, Holy Spirit University of Kaslik, P.O. Box 446, Jounieh, Lebanon; 2 College of Pharmacy, Gulf Medical University, Ajman, United Arab Emirates; 3 School of Pharmacy, Lebanese International University, Beirut, Lebanon; 4 Applied Science Research Center, Applied Science Private University, Amman, Jordan; 5 Research Department, Psychiatric Hospital of the Cross, Jal Eddib, Lebanon; 6 School of Arts and Sciences, Social and Education Sciences Department, Lebanese American University, Jbeil, Lebanon

**Keywords:** Bulimia, Mental health, Emotional dysregulation, Lebanon

## Abstract

**Background:**

Bulimia nervosa (BN) is defined as repeated episodes of binge eating, followed by compensatory behaviors such as self-induced vomiting. BN has been shown to be associated with many co-morbidities including depression and anxiety. BN has also been associated with stress, which was shown to trigger binge eating episodes in BN. Furthermore, difficulties in emotion regulation have been seen to play an important role in the psychopathology of eating disorders, including BN. Seeing that BN is the most prevalent eating disorder in Lebanon, which is linked to the stressful events the country has been through, the study aims to examine the indirect effect of emotional dysregulation on this relationship between mental health issues (stress, anxiety and depression) and bulimia nervosa among young adults. We hypothesize that difficulties in emotion regulation would have an indirect effect in the relationship between mental health and BN.

**Methods:**

This was a cross-sectional observational study, based on an online anonymous survey, which was carried out between September and December of 2020. Participants were all 18 years and above, recruited from all Lebanese governorates (*n* = 1175).

**Results:**

Difficulties in emotion regulation mediated the association between anxiety/stress/depression and bulimia. Higher mental health issues were significantly associated with more difficulties in emotion regulation; higher difficulties in emotion regulation were significantly associated with more bulimia. Finally, higher anxiety and higher stress, but not depression, were significantly and directly associated with higher bulimia.

**Conclusion:**

Results of this study could be used by mental health professional to shed light on the difficulties in emotion regulation in patients with BN and try to use therapeutic strategies to help them better regulate their emotions.

## Background

In the Diagnostic and Statistical Manual-Fifth edition (DSM-5), Bulimia Nervosa (BN) is defined as repeated episodes of binge eating, followed by compensatory behaviors to avoid weight gain [1], driven by a distorted self-image and evaluation [1]. A diagnosis of BN is made when the individual experiences these episodes at least once a week, for the duration of three months [1]. The development of BN typically occurs during adolescence and young adulthood, with the prevalence being the highest in young adults [1]. Furthermore, in the literature, BN was seen more in females than in males [1, 2].

The relationship between stress and BN, as well as theories on affect regulation and loss of food control, have been explored throughout the literature. In one theory, stress was seen to increase the risk of binge eating episodes in individuals with BN [2, 3]. These patients also report a relief from the negative thoughts after engaging in binge eating [3, 4]. Nevertheless, other theories have suggested that negative affect and stress stay the same or increase immediately following binge eating episodes [5]. Furthermore, the trade-off and escape theories suggested that negative affect and stress do not decrease, rather these individuals use binge eating episodes to distract themselves from their emotions and replace one negative affect with a more tolerable one [5, 6].

Many psychiatric co-morbidities have been shown to be associated with eating disorders, like substance abuse, depression, and anxiety disorders [7–9]. The existing literature is still inconsistent when it comes to the relationship between anxiety disorders and eating disorders, with the causal relationship between these two entities still unsure; while some researchers say that eating disorders precede anxiety disorders, others state that eating disorders are secondary to anxiety disorders [10]. However, it was suggested that the comorbidity between anxiety and eating disorders could be due to both disorders sharing distressing childhood experiences [10]. When it comes to BN, research has found that most individuals with BN are at a higher risk of developing an anxiety disorder [10, 11]. Individuals with anorexia nervosa and BN have been shown to have traits of anxiety disorders during childhood prior to developing these eating disorders [11, 12]. However, anxiety persisted even after recovery from anorexia and BN [13].

Depression is another co-morbidity that has been frequently linked to eating disorders, especially BN [14]. In fact, studies have found that depression predisposes to developing eating disorders and that eating disorders increase the risk of developing depression [15]. In a study examining the different hypotheses regarding the relationship between depression and BN, it was seen that this relationship is driven by the binge-purge cycle [16]. Negative affect and depression change throughout the cycle; one theory suggests that BN increases depression while another suggests that BN is a consequence of depression [16]. However, these theories were found to be incomplete, making the relationship between depression and BN a subject of debate. Having depression lead to an increased risk of suicide attempt in patients with BN [17].

Emotion regulation is a set of intrinsic and extrinsic behavioral, cognitive, and physiological processes that allow individuals to regulate the expression and experience of emotions [18]. The association between emotional dysregulation and eating disorders has gained interest, especially after the emergence of the emotional eating theory that highlights the fact that eating is used by individuals with eating disorders to regulate their emotions [19]. Emotions and disordered eating have been linked in multiple studies [19–21]; some report eating less when anxious [21], while others report increasing food intake when they are sad [21]. It has been seen that the higher the emotional dysregulation, the more severe the psychopathology in eating disorders [22, 23]. Individuals with eating disorders, especially BN, have been seen to have deficits in emotion regulation [20, 22, 24]. Emotion regulation has been linked to eating disorders in many aspects; in fact, theoretical models of emotion regulation reported that difficulties in emotion regulation is considered a transdiagnostic factor among all eating disorders [25]. Studies have also found that individuals with eating disorders found it difficult to differentiate between different emotions, as well as difficulties in regulation and attenuation of their emotions [25]. Furthermore, individuals with eating disorders have difficulties in emotion recognition; individuals with anorexia nervosa were found to have difficulties with labelling basic emotions when verbal cues are absent [26], while BN patients reported negative emotions preceding binge eating [27]. Moreover, some studies have found that individuals with BN have more difficulties in the impulsivity aspect of emotion regulation, as they have more difficulties in controlling their behavior in response to emotional distress [2]. In addition, emotion dysregulation, even though present in eating disorders, it differs among them; in fact, individuals with anorexia nervosa eat more when they are happy, while individuals with BN increase their food intake when they are experiencing, sadness, stress, and anxiousness [20].

Emotion regulation was seen to play an important role in the development, psychopathology, and outcome of eating disorders. Thus, the indirect effect of emotion regulation on the relationship between eating disorders and anxiety, depression and stress, has also been a subject of interest. In a study by Prefit and Szentagotai-Tătar (2018), difficulties in emotion regulation mediated the effect between eating disorders and depression [28]. Another study found that emotional dysregulation was a mediator in the relationship between dysfunctional personality traits and eating disorders [29]. In addition, emotion regulation played a mediating role on the relationship between BN and attachment related anxiety [24]. Emotional dysregulation, particularly negative affect, was also seen to mediate the relationship between stressful events and bulimic behavior [30].

Eating disorders were first thought to be most prevalent in Western countries [31], however many cultures have witnessed a rise in eating disorders [32], especially because of the prolonged exposure to the media [33]. In the Arab world, the ideals of beauty shifted over time. Culturally Arabs considered a full, curvy figure to represent the ideal standard of beauty, as it symbolizes fertility and wealth [34]. However, Arab culture shifted; adopting Western habits, as well as exposure to western media, contributed to the rise of eating disorders, which started getting reported in the Arab world around 1986 [35]. Furthermore, data on eating disorders in Arab countries and differences in eating disorders expression among cultures, is still very limited [35]. Nevertheless, some studies reported that Arabs tend to express eating disorders somatically more than psychiatrically, by reporting symptoms such as stomach aches and nausea [35].

Studies conducted on Lebanese young adults, showed a desire to become thin, food avoidance, caloric awareness [36], as well as prevalent abuse of diet pills and laxatives [37, 38]. Furthermore, Lebanese young adults were found to be prone to developing an eating disorder [39]; 46% of Lebanese individuals presenting for an eating disorder, were diagnosed with BN [40]. This rise in disordered eating in Lebanon, might be linked to the stressful events the country has been through; a study conducted during wartime and traumatic events in Lebanon, showed that the population witnessed a change in eating behavior that led to an increase in the risk of developing eating disorders [41]. Moreover, the economic and political crises faced by the Lebanese population, following the COVID-19 pandemic and the Beirut blast on August 4, 2020, were responsible for a mental health decline seen in Lebanon [42, 43].

The topic of eating disorders is still not being explored in depth in Lebanon, even though it was proven that this population is prone to developing eating disorders. Furthermore, the decline in mental health throughout the population following multiple traumatic and stressful events could make Lebanese people more vulnerable to emotional dysregulation and psychopathologies. Since BN is the most seen eating disorder in Lebanese young adults, it would be interesting to examine the indirect effect of emotional dysregulation in the relationship between other mental health issues such as anxiety, depression and stress and BN among this group. We hypothesize that there will be a positive association between mental health problems and bulimia nervosa and that difficulties in emotion regulation will have an indirect effect in these associations.

## Methods

### Study design and procedure

This was a descriptive cross-sectional observational study conducted from September through December 2020 based on an online anonymous survey. The voluntary survey was conducted on Lebanese young adults located in all Governorates of Lebanon (Beirut, Mount Lebanon, North, South, and Bekaa). To minimize interviewer risks as well as meeting lockdown restrictions enforced by the Lebanese Government, a snowball sampling method was used for the survey using online Google forms. The survey was distributed via social applications including WhatsApp, LinkedIn, and Facebook. All invited participants were above 18 years of age.

### Minimal sample size calculation

A minimal sample of 417 was deemed necessary using the formula suggested by Fritz and MacKinnon [44] to estimate the sample size: $n=\frac{L}{f2}+k+1$, where f = 0.14 for small effect size, L = 7.85 for an α error of 5% and power β = 80%, and k = 6 variables to be entered in the model.

### Questionnaire

The self-administered questionnaire used was in Arabic and required approximately 20 min to complete. The anonymity of the participants was guaranteed. The first part of the questionnaire evaluated participants sociodemographic information such as age; marital status, and educational level. In addition, the household crowding index was calculated by dividing the number of persons by the number of rooms in the house, excluding the bathroom and the kitchen; the higher the household crowding index, the lower the socioeconomic status of the family [45]. The physical activity index was calculated by multiplying the intensity by the frequency by the time of physical activity [46]. The second part of the questionnaire was composed of different scales from the following:

#### Eating Attitude Test (EAT)

The EAT, validated in Lebanon [47], is used to assess disordered food attitude, by evaluating symptoms such as preoccupation with food, binge eating, compensatory behavior, and fear of gaining weight [48] The questionnaire, that showed good internal validity and reliability [48], comprises twenty-six questions each with six response options, varying from infrequently/almost never/ never (0) to always (3). It is divided into three subscales: dieting (avoidance of fatty foods and preoccupation with thinness; items 1, 6, 7, 10, 11, 12, 14, 16, 17 and 22), bulimia and food preoccupation (items 3, 4, 9, 18, 21 and 25), and oral control (self-control over food and social pressure to gain weight; items 2, 5, 8, 13, 15, 19 and 20). A score of 20 or above indicates possible disordered eating attitudes [48]. In this study, we will only use the bulimia subscale. The McDonald’s omega values in this study were as follows: total scale (ω = 0.969), dieting (ω = 0.901), bulimia (ω = 0.861), and oral control (ω = 0.794).

#### The Difficulties in Emotion Regulation Scale (DERS-16)

It is a 16-item scale that assesses emotion regulation difficulties [49]. This scale was proven to be a valid and reliable tool [50]. Items are graded using a 5-point Likert scale. Higher scores reflect more emotion regulation difficulties. Within the scale are five subscales: clarity (lack of emotional clarity; two items), goals (difficulties engaging in goal-directed behavior; three items), impulse (impulse control difficulties; three items), non-acceptance (non-acceptance of emotional responses; three items) and strategies (limited access to effective emotion regulation strategies; five items). Higher scores indicate higher difficulties in emotion regulation. The McDonald’s omega values in this study was ω = 0.961. The validated Arabic version of the scale was used [51].

#### Lebanese Anxiety Scale (LAS-10)

The LAS-10 evaluates general anxiety among Lebanese adults [52] and adolescents [53]. It consists of 10 items rated differently: items 1 to 7 are scored from “0 = not present” to “4 = very severe” and items 8 to 10 are scored from “1 = never or almost never” to “4 = almost always”. Higher scores reflect higher anxiety. The McDonald’s omega values in this study was ω = 0.920. The LAS-10 showed high reliability as well as good internal consistency [52].

#### Beirut Distress Scale (BDS-10)

The BDS-10 [54], shown to be a reliable and valid tool [54], assesses the level of distress among Lebanese adults. It has 10 items are rated from “0 = not at all” to “3 = all of the times”. Higher scores indicate higher levels of stress. The McDonald’s omega values in this study was ω = 0.878.

#### Montgomery and Asberg Depression Rating Scale (MADRS)

The MADRS [55], that has shown high internal consistency [56], measures the severity of depressive episodes for adults. It includes 10 items scored from “0 = no abnormality” to “6 = severe”. The scale has been validated in Lebanon [57]. Higher scores show higher severity of depressive symptoms. The McDonald’s omega values in this study was ω = 0.866.

### Statistical analysis

SPSS software version 25 was used to conduct data analysis. McDonald’s omega values were calculated for the reliability of the scales (values of 0.7 or more demonstrate sufficient reliability [58]). We had no missing values in the database since all questions were required in the Google form. The normality of the bulimia score was verified via the skewness and kurtosis values varying between -2 and + 2 [59] and in samples larger than 300 [60]. A bivariate analysis using the Pearson correlation test served to assess the relationship between bulimia and other continuous variables, whereas the Student t test was used to compare two means. The PROCESS SPSS Macro v. 3.4, Model 4 [61] as used to conduct the mediation analysis; three pathways were calculated: (a) Relation between anxiety/depression/stress and DERS; (b) Relation between DERS and bulimia; (c’) and (c) Direct and total effects of the relation between anxiety/depression/stress and bulimia respectively. The mediation analysis was adjusted over all sociodemographic variables that showed a *p* < 0.25 in the bivariate analysis for the elimination of a confounding effect as much as possible. Significance was defined at *p* < 0.05.

## Results

### Sociodemographic and other characteristics of the participants

A total of 1175 participants enrolled in this study; the mean age was 25.15 ± 8.54 years, with 70.0% females. Other characteristics are summarized in Table 1.

**Table 1:** Sociodemographic and other characteristics of the participants (*n* = 1175)

Variable	N (%)
**Sex**	
Male	353 (30.0%)
Female	822 (70.0%)
**Marital status**	
Single	919 (78.2%)
Married	256 (21.8%)
**Education level**	
Secondary or less	277 (23.6%)
University	898 (76.4%)
**Eating attitudes**	
Appropriate (scores < 20)	681 (58.0%)
Inappropriate (scores ≥ 20)	494 (42.0%)
	**Mean ± SD**
**Age (in years)**	25.15 ± 8.54
**Physical activity index**	25.80 ± 19.60
**Household crowding index (persons/room)**	1.11 ± 0.62
**DERS score**	27.17 ± 16.48
**EAT bulimia**	4.02 ± 5.58
**EAT dieting**	12.63 ± 11.29
**EAT oral control**	6.16 ± 6.58
**Anxiety**	18.15 ± 8.98
**Stress**	12.83 ± 7.14
**Depression**	15.15 ± 12.11

*DERS* Difficulty in emotion regulation scale, *EAT* Eating attitude test

### Bivariate analysis

The bivariate analysis results are shown in Tables 2 and 3. A higher mean bulimia score was seen in males compared to females (0.78 vs 0.70; *p* = 0.02). Higher anxiety (*r* = 0.16), stress (*r* = 0.08) and difficulties in emotion regulation (*r* = 0.17) were significantly associated with higher bulimia.

**Table 2:** Bivariate analysis of the categorical variables associated with bulimia

Variable	Bulimia score	*P*
**Sex**		**.020**
Male	.78 ± .37	
Female	.70 ± .40	
**Marital status**		.499
Single	.71 ± .39	
Married	.74 ± .40	
**Education level**		.311
Secondary or less	.75 ± .38	
University	.71 ± .39	

Numbers in bold indicate significant *p*-values

**Table 3:** Bivariate analysis of the continuous variables associated with bulimia

	**1**	**2**	**3**	**4**	**5**	**6**	**7**	**8**
1. Bulimia	1							
2. Anxiety	.16***	1						
3. Stress	.08*	.76***	1					
4. Depression	.04	.45***	.391***	1				
5. DERS	.17***	.71***	.61***	.37***	1			
6. Age	.04	-.09**	-.07*	-.14***	-.07*	1		
7. Household crowding index	-.06	.04	.03	.03	.03	-.12***	1	
8. Physical activity index	.01	-.07*	-.06	-.03	-.03	-.06*	-.05	1

*DERS* Difficulty in emotion regulation scale. Numbers represent Pearson correlation coefficient

^***^p < 0.001; **p < 0.01; *p < 0.05

### Indirect effect analysis

**Fig. 1:**
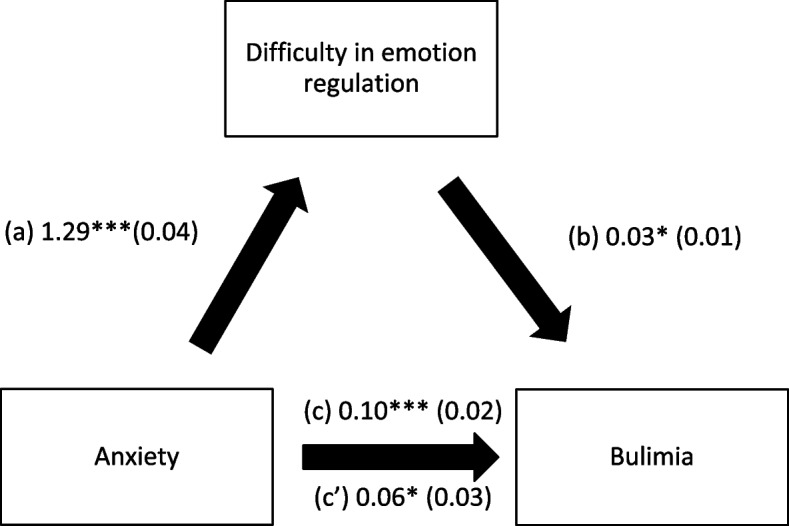
**a** Relation between anxiety and difficulty in emotion regulation (R^2^ = 49.72%); **b** Relation between difficulty in emotion regulation and bulimia (R^2^ = 4.13%); **c** Total effect of the relation between anxiety and bulimia (R.^2^ = 3.67%); (c’) Direct effect of the relation between anxiety and bulimia. Numbers are displayed as regression coefficients (standard error). ***p < 0.001; *p < 0.05

**Fig. 2:**
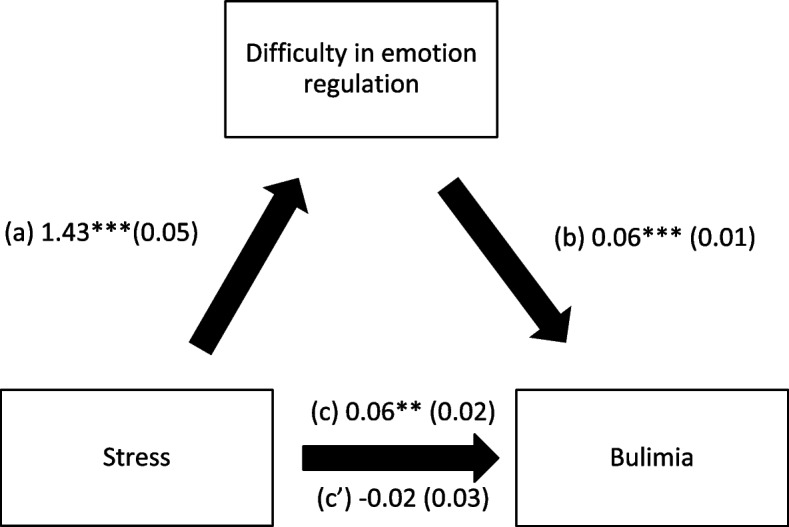
**a** Relation between stress and difficulty in emotion regulation (R^2^ = 38.15%); **b** Relation between difficulty in emotion regulation and bulimia (R^2^ = 3.73%); **c** Total effect of the relation between stress and bulimia (R.^2^ = 1.71%); (c’) Direct effect of the relation between stress and bulimia. Numbers are displayed as regression coefficients (standard error). ***p < 0.001; **p < 0.01

**Fig. 3:**
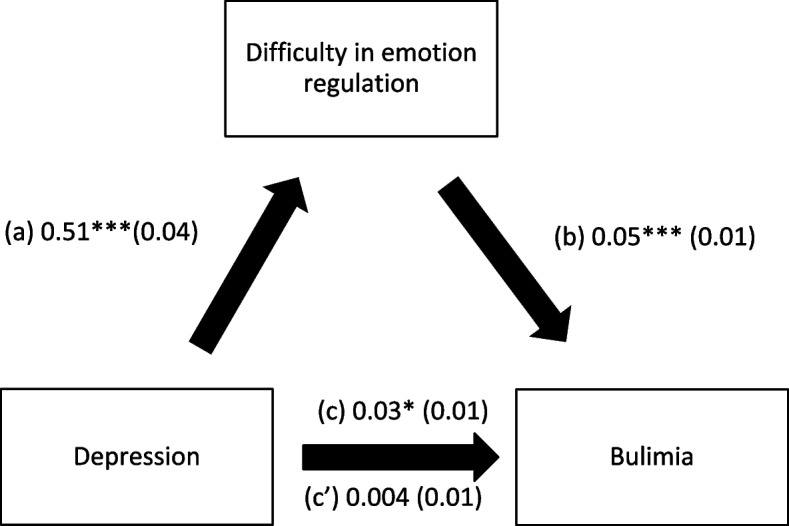
**a** Relation between depression and difficulty in emotion regulation (R^2^ = 14.03%); **b** Relation between difficulty in emotion regulation and bulimia (R^2^ = 3.68%); **c** Total effect of the relation between depression and bulimia (R.^2^ = 18.18%); (c’) Direct effect of the relation between depression and bulimia. Numbers are displayed as regression coefficients (standard error). ***p < 0.001; *p < 0.05

Difficulties in emotion regulation mediated the association between anxiety/stress/depression and bulimia (Table 4, Figs. 1, 2, and 3). Higher mental health issues were significantly associated with more difficulties in emotion regulation; higher difficulties in emotion regulation were significantly associated with more bulimia. Finally, higher anxiety and higher stress were significantly and directly associated with higher bulimia.

**Table 4:** Indirect effect analyses results, taking anxiety/depression/stress as independent variables, difficulties in emotion regulation as the mediator and bulimia as the dependent variable

Independent variable	Direct effect			Indirect effect		
	**Beta**	**SE**	***p***	**Beta**	**Boot SE**	**Boot CI**
Anxiety	.06	.03	.019	.04	.02	.01-.08^a^
Stress	-.02	.03	.415	.09	.02	.05-.12^a^
Depression	.004	.01	.794	.03	.01	.02-.04^a^

^a^indicates significant indirect effect

## Discussion

This study aimed to explore the correlates of BN in sample of Lebanese young adults, as well as the indirect effect of emotion regulation on the relationship between BN and other mental health issues, such as anxiety, depression and stress.

Higher anxiety and higher stress were seen to be significantly associated with higher BN in this study, in line with previous literature [13, 62, 63]. In trying to explain the relationship between anxiety disorders and eating disorders, it was seen that patients in both disorders have experienced early-life distressing experiences, thus developing harm avoidance cognition, leading them to disordered behavior when facing triggers [10]. Anxiety disorders traits have been associated with BN, especially compulsivity and impulsivity [64], which are seen during episodes of binge eating and compensatory behaviors. Individuals with eating disorders use dysfunctional eating as a coping mechanism [65]. However, this disordered mechanism leads to an increased negative affect and feelings of guilt [65], which might consequently lead to compensatory behaviors. Furthermore, patients with BN have been seen to have a distorted self-image [1, 66], searching for acquiring a certain body type, deemed perfect by societal and media pressure [66, 67]. In fact, overvaluation of shape and weight is one of the diagnostic criteria in BN [1]; self-evaluation in BN is influenced by body shape and weight [1]. Moreover, changes in overvaluation of shape and weight predicted the chronic persistence of BN, as well as the frequency of binge eating episodes [68]. In Lebanon, researchers reported that the most common co-morbidities for eating disorders patients were anxiety and depression [40]. Moreover, cultural differences have been a subject of interest throughout the literature when it comes to eating disorders. These disorders were previously considered higher in white women, who were thought to chase a thinner ideal than other ethnicities [69]. These cultural differences come from the fact that beauty standards differ between cultures [70, 71]. However, other studies have proven that there are more similarities between ethnicities than differences when it comes to eating disorders [72, 73]. This discordance in findings when comparing cultures, opens the door for more exploration, especially that anxiety-related body shape may vary between cultures.

The physiological response to acute and chronic stress plays a role in the pathophysiology of BN. In fact, perceived stress increases prior to binge eating episodes, which leads to a decreased activation to food cues, causing the symptoms in BN [4, 74]. Moreover, stress may trigger binge eating episodes in BN patients by decreasing brain reward response to anticipating food or eating, and brain activation in attention networks; the latter provides distraction from self-awareness and relief following binge eating [4, 74].

In this study, difficulties in emotion regulation were associated with higher bulimia, in line with previous findings that showed that difficulties in emotion regulation play a role in the psychopathology of eating disorders [20, 21]. Individuals with BN have low distress tolerance, difficulties controlling their behaviors and deficits in emotion regulation strategies [25]. Patients with BN might resort to binge eating to better regulate their emotions since negative affect increases before binge eating and decreases after it [27, 75]. Furthermore, patients with BN have lower awareness of emotions and present more emotion suppression [25, 76]. Individuals with BN struggle with difficulties regulating their emotions, and describe binge eating as a way to fill the void left by the aspect of positive emotions [77]. Negative affect experienced by BN patients, as well as their distorted self-image, promoted dietary restraints [78, 79]; it was seen that more severe dietary restraints promoted binging and purging in BN [76, 80].

In our study, higher mental health issues were significantly associated with more difficulties in emotion regulation, in agreement with previous results [81]. Emotion regulation is affected by many factors. These factors include recognition of emotions and applying adaptive strategies to face them [82]. Mental health issues were seen to affect both factors, thus leading to higher difficulties in emotion regulation [82]. Individuals with BN were seen to have deficits in emotion regulation [20, 22], with more difficult emotional regulation being associated with more severe psychopathology of the eating disorder [22]. BN patients are unable to use healthy coping methods when facing distress, because of deficits in emotion regulation, which leads them to maladaptive coping behaviors [83].

Depression was not significantly and directly associated with higher BN, which is not in line with other studies in the literature, where depression was seen to be associated with BN [14, 84]. This insignificant association may be due to the fact that in this study, a different scale was used. Another reason might be the fact that the population studied is different, which may influence predicting factors of BN. However, when mediating the effects of emotion regulation, BN was associated with depression.

The results of this study showed that difficulties in emotion regulation mediated the association between bulimia nervosa and depression/anxiety/stress. Emotion dysregulation was shown to be a mediator in the relationship between depression and disordered eating [28], between attachment related anxiety and eating disorders [24], as well as between stress and bulimic behavior [30]. Emotion dysregulation is involved in anxiety disorders and depression [81]. The dysfunctional fear response in patients with anxiety is caused by deficits in emotional awareness and acceptance, as well as coping [81], while individuals with depression have difficulties identifying negative feelings and regulating them [85]. Furthermore, patients with BN showed a tendency to avoid emotions [86], as well as an inability to regulate emotions, leading to dysfunctional eating behaviors [2]. The role of emotion regulation in mental health issues and eating disorders is still being explored in the literature, however, studies exploring the mediating effect of difficulties in emotion regulation on the relationship between mental health and bulimia nervosa are very scarce; further studies examining its mediating effect would be of interest.

### Clinical implications

Eating disorders have gained more interest across the world, especially in light of the high influence of social media that is projecting certain standards of beauty on both females and males. Bulimia nervosa is the result of dysfunctional emotion regulation and a desire to relief stress, while trying to achieve a certain body type. Results of this study could be used by mental health professional to shed light on the difficulties in emotion regulation in patients with BN and try to use therapeutic strategies to help them better regulate their emotions. Furthermore, eating disorders have been rarely studied in Arab countries, which makes this study done in Lebanon valuable to the literature, shedding light on the problem of eating disorders in this country.

### Limitations

Despite the contribution of our study to the literature regarding BN, it is important to consider some of the limitations. All data was obtained using self-report instruments, thus, it might be possible that some participants have misreported some of the questions. Moreover, some symptoms such as binge eating, and compensatory behaviors are difficult to assess via self-report tools and would require clinical interviews by mental health professionals to be adequately assessed. The study was a cross-sectional design, thus causal relations between the studied variables could not be explored. Furthermore, the Lebanese Anxiety Scale (LAS-10), used in this study assesses only general anxiety symptoms but does not address anxiety disorders, such as post-traumatic stress disorder (PTSD), that are considered to be more complexed phenomena. In addition, the study was performed during COVID-19 lockdown and confinement, which could have influenced our study results: the lockdown condition may have exacerbated psychological stress and anxiety, leading to more problematic eating. A selection bias is possible because of the snowball sampling technique used to recruit participants; thus, the results may not be generalizable. Finally, a residual confounding bias is also possible since not all factors associated with BN were considered in this paper.

## Conclusion

Emotion regulation is a scientific concept being explored more throughout the literature. Its implication in mental disorders has been investigated, but is still unclear. The current study showed that emotion regulation mediated the relationship between bulimia nervosa and mental health issues. The results of this study could be used for more awareness among mental health professionals, to shed light on difficulties in emotion regulation in patients with BN and use therapeutic strategies to help them regulate their emotions.

## Data Availability

The authors do not have the right to share any data information as per the ethics committee rules and regulations but are available upon a reasonable request from the corresponding author (SH).
